# Vitamin D assessment in perioperative medicine and critical care: A prospective observational pilot study

**DOI:** 10.1007/s00508-019-01584-x

**Published:** 2019-12-04

**Authors:** Paul Zajic, Stefan Heschl, Michael Schörghuber, Petra Srekl-Filzmaier, Tatjana Stojakovic, Viktoria Weixler, Sieglinde Zelzer, Karin Amrein

**Affiliations:** 1 Div. of General Anaesthesiology, Emergency- and Intensive Care Medicine, Dept. of Anaesthesiology and Intensive Care Medicine, Medical University of Graz, Graz, Austria; 2 Div. of Anaesthesiology for Cardiovascular and Thoracic Surgery and Intensive Care Medicine, Dept. of Anaesthesiology and Intensive Care Medicine, Medical University of Graz, Graz, Austria; 3 Div. of Cardiac Surgery, Dept. of Surgery, Medical University of Graz, Graz, Austria; 4 Clinical Institute of Medical and Chemical Laboratory Diagnostics, University Medical Centre Graz, Graz, Austria; 5 Div. of Endocrinology and Diabetology, Dept. of Internal Medicine, Medical University of Graz, Graz, Austria

**Keywords:** Vitamin D, Vitamin D deficiency, Critical care, Diagnosis

## Abstract

**Background:**

There is controversy about the impact of acute illness on vitamin D levels. This study was carried out to assess the influence of perioperative fluid loading on 25-hydroxy-vitamin D [25(OH)D] levels. The study evaluated the clinical utility of a commonly available chemiluminescence assay (ECLIA, IDS-iSYS) and liquid chromatography/mass spectrometry (LC-MS/MS) in the diagnosis of vitamin D deficiency in this setting.

**Methods:**

In this prospective observational pilot study in adult patients undergoing cardiovascular surgery on cardiopulmonary bypass (CPB), blood samples drawn at preoperative baseline (t1), after weaning from CPB (t2), on intensive care unit (ICU) admission (t3) and on the first (t4) and second (t5) postoperative days were analyzed.

**Results:**

A total of 26 patients (130 samples) were included in this study. Fluid loading by CPB led to a median reduction of 25(OH)D by −22.6% (range −54.5% to −19.5%) between t1 and t2. Cohen’s kappa (κ) for method agreement for vitamin D deficiency (tested cut-off values 20 ng/ml and 12 ng/ml), was κ = 0.291 (*p* < 0.001) and κ = 0.469 (*p* < 0.001), respectively. The mean difference between measurements by ECLIA and LC-MS/MS was 4.8 ng/ml (±5.7), Pearson’s r for correlation was 0.73 (*p* < 0.001). The biologically inactive C3-epimer did not contribute to 25(OH)D levels assessed by LC-MS/MS.

**Conclusion:**

The 25(OH)D measurements by chemiluminescence assays can noticeably deviate from those measured by LC-MS/MS, which can be considered the unequivocal gold standard. These assays may still be acceptably reliable in the screening for vitamin D deficiency, especially in the setting of low vitamin D levels. Stricter definitions, e.g. serum 25(OH)D levels lower than 12 ng/ml, may be used to diagnose deficiency with low false positive rate.

**Trial Registration:**

DRKS00009216, German Clinical Trials Registry (www.drks.de)

## Introduction

Low vitamin D status was reported to be significantly associated with a poor outcome in critically ill patients in several studies over the last decade [1–3]. These findings led to the performance of one large randomized clinical trial of high-dose vitamin D supplementation, albeit an overall negative trial, which sparked further scientific and clinical interest because a mortality benefit was found in a prespecified subgroup of patients deemed to be severely vitamin D deficient [4].

Since poor vitamin D status may contribute to excess mortality in critical illnesses, vitamin D treatment seems to be feasible, safe and possibly advantageous. As vitamin D deficiency is also highly prevalent in this setting, high-dose vitamin D treatment [5] may become an adjunctive therapy in critical care in the future; however, patient selection is important and some data suggest that the definition of deficiency may be different in this patient population [6].

Some studies in both critically ill and non-critically ill patients have raised the possibility that vitamin D status is inversely associated with inflammation and that levels may decrease rapidly during acute inflammation as well as fluid loading [7, 8]. Low vitamin D levels have been suggested to be reflective of the severity of illness in critically ill patients rather than being an independent influencing factor on outcome [9, 10]. The use of 25-hydroxyvitamin D [25(OH)D] may be unreliable in critical illness, since both 25(OH)D and serum vitamin D binding protein (VDBP) have been shown to significantly decrease during inflammation [8, 9]. Additionally, there is ongoing debate about which vitamin D metabolite is best suited to represent the vitamin D status in critically ill patients and which methods should be employed [11]. Measurement of serum 25(OH)D by LC-MS/MS has been found to be superior to the use of immunoassays in non-critically ill patients who received a fluid bolus of crystalloid solution [12].

When turnaround times should be minimal the use of automated readily available methods of 25(OH)D testing is certainly preferable to the time-consuming method of LC-MS/MS in clinical routine [13, 14]. International standardization of current 25(OH)D immunoassays has reduced previously reported interlaboratory variation [15, 16]; however, differences seem to be more prominent in specific populations, such as hemodialysis patients, critically ill individuals or pregnant women [17, 18]. Regular LC-MS methods may also detect the biologically inactive C3-epimer which may significantly contribute to 25(OH)D concentrations and can therefore lead to overestimation of 25(OH)D levels [19, 20]. The percentage of this inactive epimer seems to be higher than reported in other populations but its role in critical care is currently unknown.

## Patients, materials and methods

### Aim, design and setting

A prospective observational pilot study was performed in adult patients undergoing major cardiovascular surgery by analyzing vitamin D levels at different time points preoperatively and postoperatively using a widely available, rapid and inexpensive assay (ECLIA, chemiluminescence technology, IDS-iSYS, Immunodiagnostic Systems Holdings, Tyne & Wear, United Kingdom) and the gold standard LC-MS/MS (including detection of the C3 epimer) to confirm and quantify the decrease in vitamin D levels. The study aimed to evaluate the agreement between these methods in the diagnosis of vitamin D deficiency according to commonly used thresholds, to quantify the inactive C3 epimer and its contribution to total 25(OH)D levels, assess the impact of fluid loading and inflammation on vitamin D status and on the utility of the measurement methods in the diagnosis of vitamin D deficiency.

### Study population and inclusion criteria

Adult patients scheduled for cardiovascular surgical procedures requiring cardiopulmonary bypass (CPB, e.g. coronary artery bypass grafting, CABG, CABG plus valve surgery, double valve surgery, thoracic aortic aneurysm repair) who were deemed to have a high risk of prolonged postoperative intensive care unit (ICU) stay (48 h or longer) were included in this study. Patients undergoing elective and acute procedures were included if patients were able to give informed consent preoperatively. Patients undergoing mitral valve surgery were not included to avoid parallel inclusion in a competing trial.

### Sample collection and laboratory analysis

Blood samples were drawn as part of routine clinical interventions at predefined time points during the course of preoperative evaluation, the surgical procedure and the ICU stay. These timepoints were: preoperative baseline (t1), after weaning from CPB (t2), on admission to ICU (t3) and the mornings of the first (t4) and second (t5) postoperative days.

The 25(OH)D levels and 1,25(OH)_2_D levels were measured using an assay based on chemiluminescence technology (IDS-iSYS, Immunodiagnostic Systems). For 25(OH)D, the assay coefficients of variation for control levels are 13.4% at 13 ng/mL, 10% at 31 ng/mL, and 9.4% at 64 ng/ml. The lower detection limit of this test is 7.0 ng/ml; values below the lower detection limit were used as 3.5 ng/ml for this analysis according to convention. The performing laboratory routinely participates in the vitamin D external quality assessment scheme (DEQAS) program [16]. Routine laboratory parameters as well as parameters concerning the vitamin D axis and markers of bone metabolism were analyzed using fresh serum samples. Liquid chromatography tandem mass spectrometry (LC-MS/MS) using commercially available, standardized columns and sera (ClinMass® complete kit for vitamin D analysis, Recipe®, Munich, Germany) was used to analyze frozen samples (−70 °C) in a batch following completion of patient inclusion.

### Statistical analysis

Demographic data and indicators of physical status, relevant routine laboratory results and parameters of intervention of the study population are presented as median values ± interquartile ranges (IQR). Changes from baseline vitamin D status over the investigation period were tested using Wilcoxon rank-sum test. Relative changes in vitamin D levels over time were assessed for linear correlation with fluid balance using Pearson’s correlation coefficient. The concurrence of the investigated methods of vitamin D measurements (ECLIA immunoassay, LC-MS/MS) was examined using mean difference, Pearson’s correlation coefficient, and Bland-Altman analysis. The diagnostic utility of measurements using both methods following fluid loading and inflammation was assessed by comparison with baseline LC-MS/MS measurements as the gold standard. Generally agreed upon cut-off values for the overall population [21] as well as a potential cut-off value in critical illness derived from a previous study [4] were evaluated. All statistical analyses were performed using SPSS 25 (IBM SPSS Statistics, 2018, Armonk, NY, USA) and *p* values below 0.05 were considered significant.

## Results

A total of 26 patients providing 130 samples for analysis were included in this analysis. These patients were predominately male (*n* = 19, 73%), all of Caucasian ethnicity, with a median age of 67 years (range 60–76 years). Patients were treated in the ICU for a median of 3 days (2–6 days) and remained hospitalized for a median of 14 days (13–17 days). Both in the ICU and in the hospital there were no fatalities (*n* = 0, 0%) in this cohort (Table 1).

**Table 1:** Baseline and outcome characteristics of patients providing samples for the study (*n* = 26)

Variable		
*n of patients*		26
*Age (years) (median, IQR)*		67 (60–76)
*Male sex (n, %)*		19 (73%)
*Caucasian ethnicity (n, %)*		26 (100%)
*BMI (median, IQR)*		26 (24–28)
*ASA score (median, IQR)*		4 (3–4)
*Pre-existing conditions and treatments (n, %)*		
Chronic kidney disease		8 (31%)
Chronic hepatic disease		0 (0%)
Osteoporosis		1 (4%)
Vitamin D treatment		2 (8%)
*Type of surgery (n, %)*		
CABG		12 (46%)
AVR		4 (15%)
CABG + AVR		5 (19%)
AVR + atrial septal defect repair		1 (4%)
Aortic aneurysm repair + AVR		4 (15%)
*Length of stay (days) (median, IQR)*		
ICU		3 (2–5)
Hospital		14 (13–15)
*Hospital mortality (n, %)*		0 (0%)

*ASA score* American Society of Anesthesiologists physical status classification system, *AVR* aortic valve replacement, *BMI* body mass index, *CABG* coronary artery bypass grafting, *ICU* intensive care unit, *IQR* interquartile range

Patients were treated according to local protocol based on clinical needs as assessed by the treating physicians. Parameters of physical status, relevant routine laboratory measurements and fluid balance are shown in Table 2.

**Table 2:** Fluid balance, markers of inflammation and measures of the vitamin D axis at the five observation time points of the study (t1–t5); depicted are median (IQR)

	t1	t2	t3	t4	t5
Fluid balance (ml)	–	3700 (3100–4111)	920 (−535–1378)	1895 (1265–3038)	124 (−201–825)
Hematocrit (%)	35.5 (31.0–38.4)	26.2 (25.1–28.7)	31.0 (26.0–34.4)	29.5 (25.6–32.9)	27.8 (24.8–30.4)
Leukocytes (G/l)	4.8 (4.2–5.8)	7.2 (6.0–10.4)	9.7 (6.4–12.1)	10.3 (8.2–12.2)	11.7 (9.7–12.7)
CRP (mg/l)	1.3 (0.7–2.3)	1.1 (0.6–2.0)	2.0 (0.7–4.6)	63.4 (50.4–77.6)	146.0 (92.4–203.5)
Albumin (g/dl)	3.5 (3.3–3.7)	2.4 (2.2–2.7)	2.8 (2.4–3.0)	3.0 (2.8–3.3)	2.9 (2.6–3.1)
Total protein (g/dl)	6.0 (5.7–6.3)	4.3 (3.7–4.6)	4.7 (4.2–5.0)	5.1 (4.6–5.5)	5.2 (4.9–5.7)
PTH (pg/ml)	48.2 (30.6–60.1)	29.1 (21.2–44.0)	39.8 (23.3–55.7)	44.6 (28.0–70.2)	51.1 (33.8–73.5)
Ca^2+^, total (mmol/l)	2.29 (2.20–2.34)	2.11 (2.06–2.20)	2.20 (2.08–2.25)	2.12 (2.03–2.23)	2.08 (2.00–2.14)
Ca^2+^, ionized (mmol/l)	1.18 (1.15–1.20)	1.19 (1.17–1.23)	1.18 (1.16–1.27)	1.11 (1.06–1.15)	1.10 (1.05–1.14)
1,25(OH)_2_D (pmol/l)	76 (36–89)	34 (21–51)	35 (19–54)	34 (23–64)	29 (18–64)

*1,25(OH)2D* 1,25-dihydroxyvitamin D, *CRP* C-reactive protein, *PTH* parathyroid hormone, *Ca*^*2+*^ calcium, *IQR* interquartile range

### Vitamin D trends

**Fig. 1:**
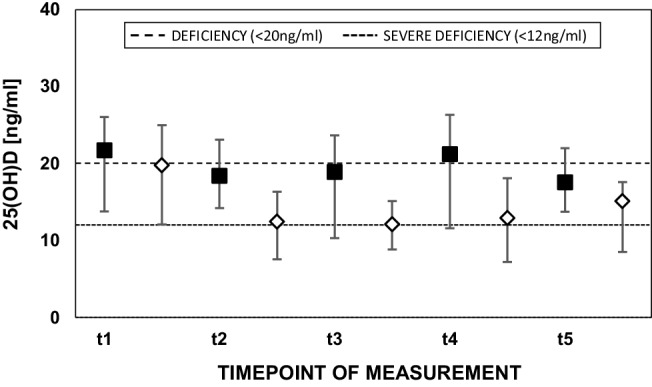
Median (interquartile range) 25(OH)D measurements by ECLIA (*black squares*) and LC-MS/MS (*white diamonds*) at preoperative baseline (t1), after the end of cardiopulmonary bypass (t2), on admission to ICU (t3), and on the first (t4) and second (t5) postoperative day. *25(OH)D* 25-hydroxyvitamin D, *ECLIA* electrochemiluminescence assay, *LC-MS/MS* liquid chromatography-mass spectroscopy/mass spectrometry

The median 25(OH)D level at baseline (t1) measured by the chemiluminescence assay was 21.7 ng/ml (13.8–26.0 ng/ml), which dropped to 18.4 ng/ml (14.2–23.1 ng/ml, *p* = 0.15 for comparison with t1) over the course of cardiopulmonary bypass (t2). On ICU admission (t3) median 25(OH)D level was 18.9 ng/ml (10.3–23.7 ng/ml), still lower than at baseline (*p* = 0.09). On the first postoperative day (t4), median 25(OH)D level was 21.2 ng/ml (11.6–26.3 ng/ml *p* = 0.29). On the second postoperative day (t5), median level was at 17.6 ng/ml (13.8–22.0 ng/ml, *p* = 0.16) (Fig. 1).

When measured by mass spectrometry the median 25(OH)D at t1 was 19.8 ng/ml (12.1–25.0 ng/ml). There was a marked drop following fluid loading by CPB until t2 (12.5 ng/ml, 7.5–16.3 ng/ml, *p* = 0.001). Measurements following ICU admission and intensive care treatment were 12.1 ng/ml (8.8–15.1 ng/ml, *p* < 0.001) at t3, 12.9 ng/ml (7.2–18.1 ng/ml, *p* = 0.004) at t4 and 15.1 ng/ml (8.5–17.6 ng/ml, *p* < 0.001) at t5 (Fig. 1).

### Effect of fluid loading

Fluid loading during CPB for surgery and maintenance of homeostasis during intensive care led to noticeable changes in 25(OH)D levels. There was, however, considerable variation in both the magnitude of these changes and their detection by the investigated measurement methods. Using LC-MS/MS, median relative changes in 25(OH)D levels were −22.6% (−54.5% to −19.5%) between t1 and t2, +1.5% (−27.3% to 18.2%) between t2 and t3, +15.2% (−3.3% to 32.9%) between t3 and t4 and +7.8% (−5.3% to 27.3%) between t4 and t5. In comparison, using the chemiluminescence assay, relative changes were ±0.0% (−28.6% to +7.9%) between t1 and t2, +12.4% (−12.8% to +25.3%) between t2 and t3, +17.2% (−0.1% to +29.7%) between t3 and t4, and −0.7% (−39.2% to +9.4%) between t4 and t5. Due to this variation, no linear correlation between relative 25(OH)D levels and fluid balance could be found for measurements by ECLIA; Pearson’s correlation coefficient was −0.019 (*p* = 0.87). There was a weakly significant correlation between LC-MS/MS measurements and fluid balance; Pearson’s r was −0.28 (*p* = 0.03). Correlation between fluid balance and 25(OH)D was similarly poor at individual timepoints; Pearson’s r for ECLIA and fluid balance were: 0.00 (*p* = 0.99) at t2, 0.29 (*p* = 0.14) at t3, −0.16 (*p* = 0.49) at t4 and 0.22 (*p* = 0.36) at t5; Pearson’s r for LC-MS/MS and fluid balance were: −0.42 (*p* = 0.14) at t2, 0.01 (*p* = 0.97) at t3, −0.32 (*p* = 0.24) at t4 and 0.13 (*p* = 0.62) at t5.

Hemodilution due to fluid loading had noticeable influences on other parameters of the vitamin D axis as well. Median (IQR) 1,25(OH)_2_D levels were 76 pmol/l (36–89 pmol/l) at t1 and dropped to 34 pmol/l (21–51 pmol/l) at t2. No return to baseline was observed during the study duration; further median (IQR) 1,25(OH)2D levels were 35 pmol/l (19–54 pmol/l) at t3, 34 pmol/l (23–64 pmol/l) at t4 and 29 pmol/l (18–64 pmol/l) at t5. Parathyroid hormone (PTH) was also affected by fluid loading; median PTH levels dropped from 48.2 pg/ml (30.6–60.1 pg/ml) at t1 to 29.1 pg/mol (21.2–44.0 pg/mol) at t2. No compensatory overshoot was found; further median PTH levels were 39.8 pg/ml (23.3–55.7 pg/ml) at t3, 44.6 pg/ml (28.0–70.2 pg/ml) at t4 and 51.1 (33.8–73.5 pg/ml) at t5.

### Measurement methods

**Fig. 2:**
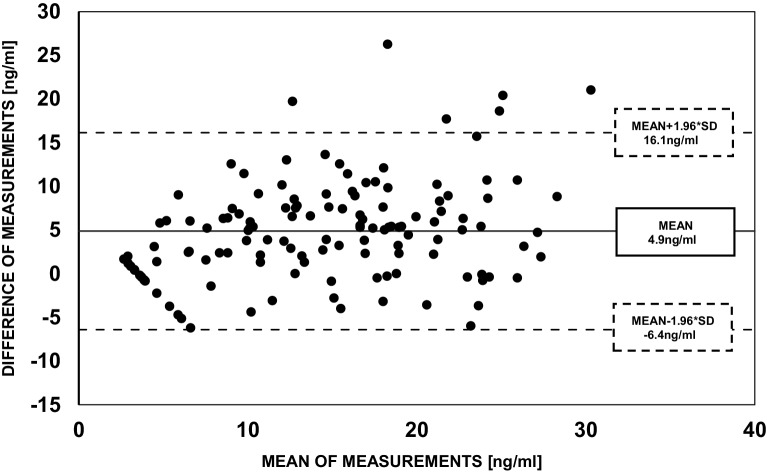
Bland-Altman plot for 25(OH)D measurements by ECLIA and LC-MS/MS. Mean difference = 4.8 ng/ml (SD ±5.7 ng/ml). *25(OH)D* 25-hydroxyvitamin D, *ECLIA* electrochemiluminescence assay, *LC-MS/MS* liquid chromatography-mass spectroscopy/mass spectrometry, *SD* standard deviation

There were noticeable differences between the 25(OH)D levels measured by ECLIA and LC-MS/MS. Numerical agreement between the methods assessed by linear correlation was acceptable, Pearson’s r was 0.73 (*p* < 0.001). Mean difference between ECLIA and LC-MS/MS was 4.8 ng/ml (±5.7). Bland-Altman analysis comparing the two methods of measurement is provided in Fig. 2.

Agreement between the two methods was limited with respect to the categorical diagnosis of vitamin D deficiency. Using cut-off values of 20 ng/ml and 12 ng/ml, the 2 methods agreed in 91 (70%) samples (Cohen’s κ 0.291, *p* < 0.001) and 95 (73%) samples (Cohen’s κ 0.469, *p* < 0.001), respectively. No epimers of 25(OH)D were found in any samples; the biologically inactive C3-epimer did therefore not contribute to the vitamin D levels assessed by LC-MS/MS.

### Diagnostic use and utility

Diagnostic characteristics of both measurement methods were assessed in intraoperative and postoperative samples, i.e. those from time points t2–t5 (*n* = 104). A 25(OH)D level below 20 ng/ml measured by LC-MS/MS at t1 was used as the gold standard for comparison. The use of 20 ng/ml as a cut-off following fluid loading would lead to low specificity, i.e. high rates of over-diagnosis, with both ECLIA and LC-MS/MS (specificity 63% and 13%, respectively). A cut-off value of 12/ng/ml means low sensitivity, but high specificity (98% and 85% for ECLIA and LC-MS/MS, respectively). Detailed results are listed in Table 3.

**Table 3:** Diagnostic characteristics of different 25(OH)D cut-off values and measurement methods in intraoperative and postoperative samples (*n* = 104) compared to their respective baselines; gold standard: preoperative vitamin D deficiency [25(OH)D < 20 ng/ml] measured by LC-MS/MS

	Sensitivity (%)	Specificity (%)	PPV (%)	NPV (%)
*ECLIA*				
25(OH)D < 20 ng/ml	73	63	76	60
25(OH)D < 12 ng/ml	38	98	96	49
*LC-MS/MS*				
25(OH)D < 20 ng/ml	98	13	64	83
25(OH)D < 12 ng/ml	72	85	89	65

*PPV* positive predictive value, *NPV* negative predictive value

## Discussion

In this study it was demonstrated that changes during the perioperative phase and critical illness significantly altered both the findings of a commonly used chemiluminescence assay for vitamin D measurements and mass spectrometry. Acute fluid loading by CPB leads to a reduction in 25(OH)D levels of about one fifth of baseline values on an individual patient level. This is somewhat lower than in a previous small study in patients undergoing major cardiac surgery [7] and other studies in non-critically ill patients [8], which suggested a reduction up to 40% of baseline levels. While this reduction in serum 25(OH)D levels is physiologically plausible following a single significant volume expansion (median 3.7l, IQR 3.1–4.1l) by CPB, interindividual variation in response was surprisingly high. Inference of premorbid vitamin D status from values measured later on therefore seems improper, especially in critically ill patients, whose volume status may be influenced by far more sources than one fluid bolus alone. This is confirmed by limited linear correlations between fluid balance as well as markers of inflammation and relative changes in 25(OH)D levels in this study. These alterations in vitamin D status in states of critical illness may contribute to overdiagnosis and potentially overtreatment later on. While supplementation of cholecalciferol during intensive care can be considered standard of care using doses recommended for the overall population (600–4000 IU daily), high bolus doses have been shown to be necessary in critical illness if correction of vitamin D deficiency is the goal [22]. In the only large randomized clinical trial investigating this intervention published to date, no significant benefit was found regarding the primary endpoint (length of stay) or overall mortality [4]. There was, however, a statistically significant and clinically potentially highly relevant reduction in mortality in severe vitamin D deficiency (*n* = 200/42% of the total population, 25(OH)D levels < 12 ng/ml).

More specific patient selection is a possible explanation for this subgroup finding. Selecting patients that benefit most from interventions both in clinical trials and routine practise is considered ever more important in times of “precision medicine” [6, 23]. This alternative cut-off for diagnosis of vitamin D deficiency was therefore tested in critically ill patients after fluid loading in this study. As could be expected from a narrower definition of the condition in question, the lower cut-off was indeed more specific for the diagnosis of vitamin D deficiency when preoperative 25(OH)D levels lower than 20 ng/ml measured by LC-MS/MS were used as the gold standard for comparison. Conversely, the rate of false negative findings would also be higher.

Readily available, economically reasonable and sufficiently specific tests are preferable both in everyday clinical practice and for conducting clinical trials to maximize the potential benefit of an intervention yet to be proven, such as high-dose vitamin D supplementation in critically ill patients. The reliability of a commercially available chemiluminescence assay (ECLIA) compared to mass spectrometry, the gold standard in this field was therefore assessed. Although the detection of the biologically inactive C3 epimer was claimed to lead to overestimation of vitamin D levels by LC-MS/MS, the findings refute this notion as no epimers were detected by LC-MS/MS, further corroborating its standing as the gold standard method.

### Strengths and limitations

This study is obviously limited by the sample size. Recruitment of a larger patient cohort was not possible for logistic reasons. Furthermore, fluid loading by CPB for cardiac surgery is not necessarily the same as fluid loading for purposes such as hemodynamic resuscitation for sepsis in intensive care. The findings presented in this study do, however, add knowledge relevant both for everyday clinical practice and scientific purposes that may be used as the basis for the definition of vitamin D deficiency as a treatment and study target.

## Conclusion

Diagnosis of vitamin D deficiency in the status of fluid loading and inflammation, such as the perioperative period and critical illness, can be complicated by hemodilution. Inference of baseline values from downstream measurements is not reliably possible. Stricter definitions, such as serum 25(OH)D levels lower than 12 ng/ml may therefore be used to diagnose vitamin D deficiency with a low false positive rate. Measurements by commonly available chemiluminescence assays may deviate noticeably from the mass spectrometry gold standard; however, they are acceptably reliable in the acute setting when vitamin D deficiency has to be diagnosed.

## Abbreviations

1,25(OH)_2_D: 1,25-Dihydroxyvitamin D
25(OH)D: 25-Hydroxyvitamin D
AVR: Aortic valve replacement
BMI: Body mass index
CABG: Coronary artery bypass grafting
ECLIA: Electrochemiluminescence assay
IQR: Interquartile range
LC-MS/MS: Liquid chromatography-mass spectroscopy/mass spectrometry
VDBP: Vitamin D binding protein
